# Population and function analysis of cultivable bacteria associated with spores of arbuscular mycorrhizal fungus *Gigaspora margarita*

**DOI:** 10.1007/s13205-017-0612-1

**Published:** 2017-04-08

**Authors:** Liangkun Long, Qunying Lin, Qing Yao, Honghui Zhu

**Affiliations:** 1 College of Chemical Engineering, Nanjing Forestry University, Nanjing, 210037 China; 2 Nanjing Institute for the Comprehensive Utilization of Wild Plants, Nanjing, 210042 China; 3 College of Horticulture, South China Agricultural University, Guangzhou, 510642 China; 4 Guangdong Institute of Microbiology, Guangdong Provincial Key Laboratory of Microbial Culture Collection and Application, Guangdong Open Laboratory of Applied Microbiology, State Key Laboratory of Applied Microbiology South China, Guangzhou, 510070 China

**Keywords:** Fungal spores, Cultivable bacteria, Stimulation of spore germination, Plant growth promotion

## Abstract

This study was aimed to investigate the diversity and function of bacterial population associated with *Gigaspora margarita* spores. The fungus was propagated in sterilized sand/soil pots using alfalfa (*Medicago sativa*), grain sorghum (*Sorghum bicolor*), or maize (*Zea mays*) as host plants, or in sterilized vermiculite pots using alfalfa as host plants, respectively. Bacteria were isolated from the new-formed spores using diluted plate method, and typical bacterial isolates were identified according to 16S rRNA gene phylogenetic analysis. Total 43 bacterial isolates affiliated to three phyla and 23 genera were obtained. The spore-associated bacterial communities were obviously different among the four source spores, suggesting that plant species or substrates could influence the bacterial population. *Bacillus* and *Streptomyces* were most frequently associated with the fungal spores. Function analysis of these bacteria by plate tests, it was found that about 30.2% isolates stimulated the spore germination, five out of seven tested isolates improved the hyphal growth, total 57.5% of the tested isolates solubilized phosphorus at different levels, 15% isolates degraded chitin, and a few isolates suppressed the growth of *Escherichia coli* or *Staphylococcus aureus*. In pot experiment, three bacterial isolates (belonging to *Curtobacterium*, *Ensifer,* or *Bacillus*, respectively) displayed improvement effect on alfalfa growth and/or the colonization of roots by *G. margarita*.

**Electronic supplementary material:**

The online version of this article (doi:10.1007/s13205-017-0612-1) contains supplementary material, which is available to authorized users.

## Introduction

Arbuscular mycorrhizal fungi (AMF) are important symbiotic partner of most terrestrial plant species. Soil bacteria selectively associated with AMF could possibly serve as the third partner of the mycorrhizal symbiosis (Levy et al. 2009; Lecomte et al. 2011; Bidondo et al. 2016). Several of these bacteria displayed beneficial effects on the performance of AMF, including the promotion of fungal spore germination, hyphal growth, the colonization of roots, and the protein expression and lipid profile (Garbaye 1994; Hildebrandt et al. 2002; Salvioli et al. 2010). Information about AMF-associated bacterial communities and their possible functions is necessary for understanding the ecological roles of the bacteria and revealing mycorrhizal ecosystem. The spore-associated bacteria (SAB) of *Gigaspora margarita* MAFF 520054 were analyzed with PCR-DGGE method, and indicated that host plants or substrates could influence the bacterial composition (Long et al. 2008). In this study, the bacterial populations associated with the fungal spores were investigated by cultured method and potential functions of these bacteria were analyzed.

## Materials and methods

### Biomaterials

*Gigaspora margarita* MAFF 520054 (from the Ministry of Agriculture, Forestry, and Fisheries Gene bank, Tsukuba, Japan) was maintained as pure propagation in our laboratory by subculturing in sand/soil pots using white cover (*Trifolium repens*) as host plant. In this study, *G. margarita* MAFF 520054 was propagated by four “plant-substrate” systems, respectively, namely, sterilized sand/soil mixture (1:1, v/v) pots combined with alfalfa (*Medicago sativa*), grain sorghum (*Sorghum bicolor*) or maize (*Zea mays*), and sterilized vermiculite combined with alfalfa. The pot experiments were carried out in a greenhouse. One-liter plastic pots were filled with the substrates, and the substrate moisture was kept at about 60% (v/v) during plant growth. In each pot, about 10 g of AMF inoculum and ten (for alfalfa) or two (for grain sorghum or maize) plant seeds were used, and 0.1 l modified Hoaglands solution (macro-elements were adjusted to 1/10 strength) was watered every 2 weeks. After approximately 4 months, the substrates mixed with AMF propagations and host roots were air dried, and the fungal spores were collected from the mixture immediately by wet sieving as previously reported (Long et al. 2008). Spores were stored under 4 °C and subsequently conducted to the isolation of spore-associated bacteria (SAB) as soon as possible.

### Isolation and identification of SAB

For each “plant-substrate” treatment, fitly mature and healthy *G. margarita* spores were used to isolate SAB. The fungal spores were put into a 1.5-ml Eppendorf tube, and washed three times with sterilized 0.75% NaCl solution. Then, the spores were suspended in 100 μl of 0.75% NaCl solution and crushed with a sterilized mini-pestle. Three aliquots of tenfold or 10^2^-fold dilutions of each fraction suspension were spread on Tryptic-Soy agar (TSA) medium. The bacterial CFU (colony formed unit) per plate was counted, and bacterial colonies representing different morphology were numbered and picked out after incubated at 26 °C for 4 days. To identify the bacteria, genomic DNA of each isolate was prepared by boiled method (Araújo et al. 2004) or lysozyme-SDS method (Rivera et al. 2003). Then, the 16S rRNA genes were PCR amplified with primer 27f (5′-agagtttgatcctggctcag-3′) or 338f (5′-actcctacgggaggcagcag-3′) paired with primer 1541r (5′-aaggaggtgatccagccgca-3′) (Brosius et al. 1981; Cocolin et al. 2001), respectively. The amplified DNA fragments were sequenced by Invitrogen Biotechnologies Co. Ltd (Shanghai, China), and the target sequences were analyzed using BLAST on the NCBI web (http://www.ncbi.nim.nih.gov/). The related sequences were collected and aligned using the ClustalX 1.83 software, and phylogenetic trees were constructed using the neighbor-joining method (Kimura’s correction model) with the Mega 4 software. The topology of the distance tree was tested by resampling data with 1000 bootstraps to provide confidence estimates.

### Effect of SAB on fungal spore germination and hyphal growth

To investigate the effect of bacterial isolates on germination of the fungal spore, each isolate was suspended with sterilized ddH_2_O up to 10^8^ cells per milliliter after cultured on TSA tube slant at 26 °C for 48 h. Surface-decontaminated *G. margarita* spores were prepared with reported method (Karandashov et al. 2000). The ‘clean’ spores (*n* = 15–25) were immerged into the bacterial suspension for 10 min at room temperature and transferred to a 1% water agar (WA) plate with a soft tweezer, then incubated at 26 °C for 14 days. Spore germination was detected under a dissection microscope, and it was recorded as germination when the tube length exceeded the spore diameter.

To assess the effect of SAB on hyphal growth of the fungus, seven isolates were selected and the same co-incubation of SAB with the fungal spores was conducted as described above. Per 15 ‘clean’ spores were used as a group, and treatment contained no bacterial cells was conducted as control. Four replications were established for each treatment. After incubation for 15 days at 26 °C, hyphal elongation (accurate to mm) of the germinated spores was assessed by the 2 mm grid method (Bécard et al. 1992).

### Phosphate solubilization, chitin degradation, and antimicrobial effects of SAB

GL medium (glucose 10 g, yeast extraction 2 g, 10% CaCl_2_ solution 10 ml, 10% K_2_HPO_4_ solution 10 ml, ddH_2_O 1000 ml, pH 7.0) plates contained CaHPO_4_ were prepared according to the document method (Cruz et al. 2008). Per five SAB isolates as a group were inoculated onto a GL plate with an inoculation loop, and cultured at 26 °C for 3–4 days. Transparent zone produced around the colony was measured with a ruler, and the corresponding isolate was recorded as a P-solubilizing bacterium. The same method was used to assess chitin-decomposing activity of SAB replacing GL medium by chitin medium (colloidal chitin 15 g, yeast extraction 3 g, (NH_4_)_2_SO_4_ 1 g, K_2_HPO_4_ 1.36 g, MgSO_4_·5H_2_O 0.3 g, ddH_2_O 1000 ml, pH 7.0). At the same time, *Escherichia coli*, *Staphylococcus aureus,* and *Fusarium oxysporum* were used to assess antimicrobial effects of the bacterial isolates with the reported methods (El-Sayed et al. 2014).

### Effect of SAB on fungal colonization and plant growth

Autoclaved sand/soil mixture at ratio of 1:1 (v/v) was used as substrates (organic matter 0.42%, N 65.2 mg/kg, P 27.3 mg/kg, K 15.7 mg/kg, Ca 101.0 mg/kg, pH 6.9) in pot experiment. For each isolate, the bacterial suspension (about 5.0 × 10^8^ cells per milliliter) was prepared as described above. Healthy and surface decontaminated *G. margarita* spores were immerged into the bacterial suspension for 10 min at room temperature. In a pot (8 cm × 10 cm) containing 320 g substrates, two alfalfa seeds were planted and inoculated with five treated fungal spores and 5 ml of the corresponding bacterial suspension. Five replications were carried out for each treatment. Plant management was conducted as described above. After 4-month growth, plants were harvested and roots were washed and isolated from the substrates. Fresh and dry weights of plant shoots were recorded immediately on harvesting and after drying at 70 °C for 24 h. Random samples of fresh roots at 0.5 g from every treatment were stained with trypan blue and examined under a microscope. Rate of AMF colonization in roots was measured by the reported method (Mcgonigle et al. 1990).

### Statistical analysis

For data analysis, one-way ANOVA was performed with the SPSS v17.0 software using Duncan’s Multiple Range Test (DMRT). A *P* value of less than 0.05 was considered statistically significant.

## Results and discussion

### Cultivable bacteria associated with *G. margarita* spores

Fungal spores were collected from the four “plant-substrate” systems, namely, from alfalfa in sand/soil pots (AS), alfalfa in vermiculite pots (AV), grain sorghum in sand/soil pots (GS), and maize in sand/soil pots (MS), respectively. Average numbers at 60, 172, 65, and 53 bacterial CFU per spore were isolated from the new-formed spores in AS, AV, GS, and MS groups, respectively (Supplementary Figure S1). More than two times of cultivable bacteria isolated from the spores in AV group compared to AS group, suggesting that vermiculite with better air permeability than sand/soil was beneficial to the breeding of bacteria. According to morphology differentia, total 43 bacterial isolates were selected and purified in the isolation experiments using four different spores (Table 1).

**Table 1:** Taxonomic positions and the diversity of the spore-associated bacteria of *G. margarita*

Phylum	Genus	Total	Source environment of the spores			
			AS	AV	GS	MS
Proteobacteria (12 genera)	*Achromobacter*	1	0	0	1	0
	*Aquitalea*	5	0	5	0	0
	*Bosea*	1	0	0	1	0
	*Burkholderia*	1	0	0	1	0
	*Cupriavidus*	3	0	0	0	3
	*Ensifer*	1	1	0	0	0
	*Lysobacter*	1	0	0	1	0
	*Mitsuaria*	1	1	0	0	0
	*Proteus*	1	0	0	1	0
	*Pseudomonas*	1	1	0	0	0
	*Ralstonia*	1	0	1	0	0
	*Rhizobium*	1	0	0	0	1
Actinobacteria (8 genera)	*Amycolatopsis*	1	0	0	0	1
	*Arthrobacter*	2	0	2	0	0
	*Curtobacterium*	1	0	1	0	0
	*Gordonia*	1	0	1	0	0
	*Leifsonia*	3	3	0	0	0
	*Mycobacterium*	1	0	1	0	0
	*Nocardia*	2	0	0	1	1
	*Streptomyces*	4	2	0	1	1
Firmicutes (3 genera)	*Bacillus*	5	2	0	1	2
	*Brevibacillus*	1	0	0	0	1
	*Paenibacillus*	4	0	0	2	2

*AS* alfalfa in sand/soil pot, *AV* alfalfa in vermiculite pot. *GS* grain sorghum in sand/soil pot, *MS* maize in sand/soil pot

By the phylogenetic analysis of the 16S rRNA genes (accession numbers: EU072704 to EU072717 and EU589400 to EU589429) of these isolates, they were affiliated to 3 phyla and 23 genera (Supplementary Figures S2a–S2c). Total 18 isolates were affiliated to 12 genera in the phylum of Proteobacteria, 15 isolates were affiliated to 8 genera in the phylum of actinobacteria, and the rest of 10 bacterial isolates were contained in 3 genera of Firmicutes (Table 1). The bacteria belonging to these phyla were also found on the spores of other AMF species, including *Funneliformis caledonium*, *Racocetra alborosea,* and *Funneliformis mosseae* (Selvakumar et al. 2016). It was showed that most isolates from the fungal spores propagated under different host plant or substrate environments were affiliated to different genera. No common genus was isolated from the groups of AS and AV, which contained the same alfalfa plants in sand/soil or vermiculite, respectively (Table 1; Supplementary Figures S2a–S2c). It was indicated that different chemical or physical conditions of the two substrates led to proliferation of distinctly different bacterial populations. The most isolates in AS group were different with those in GS or MS group which only differentiated in plant species. Obviously, host plant species also influenced the bacterial population structures. The results were consistent with the previous studies based on the molecular communication analysis of the SAB of *G. margarita* and *Gigaspora rosea* (Long et al. 2008). Four of ten isolates belonging to *Streptomyces* or *Bacillus* in AS group were simultaneously found in GS and MS groups which used different host plants in the same sand/soil pots. In addition, the common genera between GS and MS groups were *Nocardia*, *Streptomyces*, *Bacillus,* and *Paenibacillus* (Table 1). These data indicated that some bacterial genera developed specific interactions with AMF, as previously reported (Scheublin et al. 2010; Lecomte et al. 2011).

### Function analysis of SAB in plate tests

**Fig. 1:**
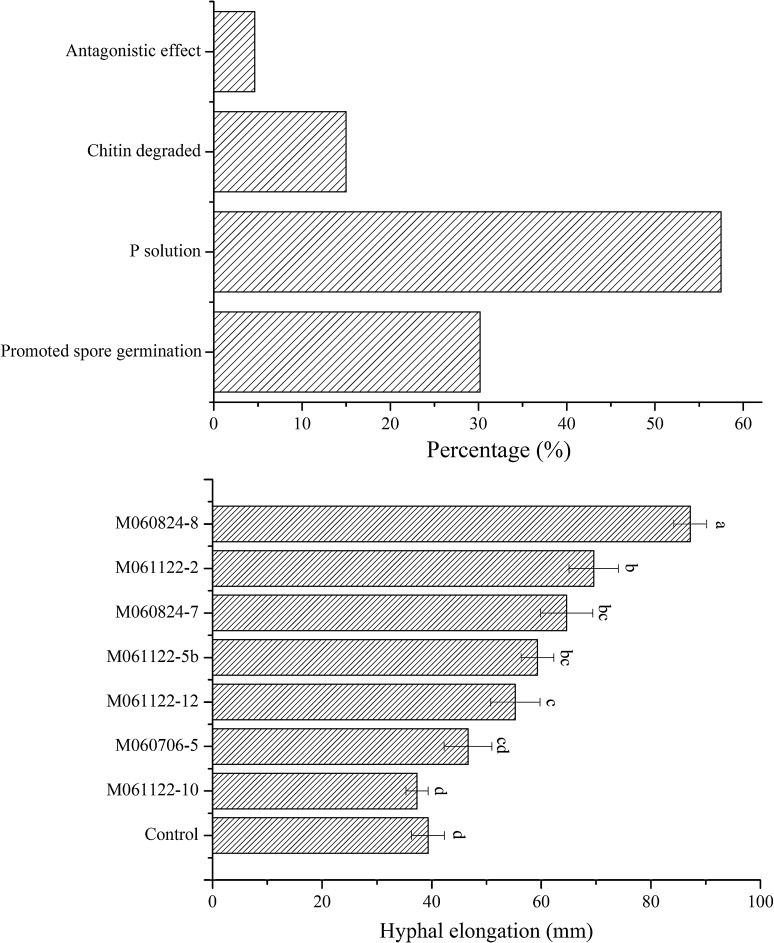
Functional evaluation of bacteria associated with the spores of *G. margarita*. **a** Percentages of bacterial isolates displaying different functions in plate tests. Total 43 isolates were tested, and the detailed records were listed in Supplementary Table S5. **b** Effect of spore-associated bacteria on in vitro hyphal growth of *G. margarita* after 15 days culture. Strains M060706-5, M060824-7, M060824-8, M061122-2, M061122-5b, M061122-10, and M061122-12 were mostly related to *Ensifer adhaerens*, *Curtobacterium luteum, Mycobacterium mucogenicum*, *Paenibacillus glycanilyticus*, *Cupriavidus pauculus*, *Bacillus bataviensis,* and *Brevibacillus agri*, respectively. Each data is the average of four replications. *Different letters* above the *error bars* indicate significant differences by DMRT test at *P* < 0.05

According to the results of plate tests, about 30.2% isolates promoted (Fig. 1a) and 11.6% isolates inhibited the spore germination, while 58.1% isolates had no significant effect on the germination (Supplementary Tables S1–S5). It was found that 57.5% tested isolates (three could not grow on the test plate) solubilized phosphorus at different levels (Fig. 1a; Supplementary Table S5). P-solubilizing bacteria were easily isolated from AMF (Taktek et al. 2015), suggesting their roles in improvement of P uptake by AMF or root hair. Previous studies revealed that some AMF-associated bacterial isolates could degrade chitin, which is one of main components of AMF spore walls, and thereby improved fungal spore germination (Ames et al. 1989; Selvakumar et al. 2016). In our study, six chitin-decomposing bacteria were isolated, and they belong to *Mitsuaria* (M060706-1b), *Streptomyces* (M060706-9), *Curtobacterium* (M060824-7), *Paenibacillus* (M061017-6), *Paenibacillus* (M061122-6), and *Streptomyces* (M061122-7), respectively (Fig. 1a; Supplementary Table S5). These data provided an additional proof for the speculation. In addition, only 4.65% isolates suppressed the growth of *E. coli* or *S. aureus*, and no one could suppress *F. oxysporum* in our test (Fig. 1a; Supplementary Table S5). It was speculated that antimicrobial activity was not the main function of the SAB from *G. margarita* MAFF 520054.

The bacteria associated with AMF or the endophytic bacteria of plant could improve the spore germination and hyphal growth of *Glomus clarum* and/or *Glomus intraradices* (Xavier and Germida 2003; Sundram et al. 2011). In this research, total seven isolates belonging to different genera and displaying improvement effect (except M061122-12) on spore germination were picked out to assess the effect on hyphal growth. It was found that the seven tested isolates except M060706-5 and M061122-10 showed positive effect on the hyphal elongation, including the isolate M061122-12 which could not promote the fungal spore germination (Fig. 1b; Supplementary Table S5). The hyphal length treated with isolate M060824-8 (the mostly related to *Mycobacterium mucogenicum*) was 2.2 times of the control group. The results indicated that stimulation of fungal growth was an important function of bacteria associated with AMF.

### Improvement of AMF colonization and plant growth by SAB

Seven bacterial isolates were selected to co-inoculate alfalfa with *G. margarita*. It was shown that three isolates (strains M060706-5, M060824-7, or M061122-10) significantly improved the fungal colonization and/or the plant growth (Table 2). The fresh weight of alfalfa roots treated with isolate M060706-5 (the mostly related to *Ensifer adhaerens*) was 1.58 times of the control group. The fresh or dry weights of plant shoot treated with isolate M060824-7 (the mostly related to *Curtobacterium luteum*) had 0.37 or 0.36 time increment compared to non-inoculated group. At the same time, inoculation with isolates M060706-5 or M061122-10 (the mostly related to *Bacillus bataviensis*) led to the increment of fungal colonization by 47 or 48%, respectively (Table 2). Previous studies also showed that bacteria associated with AMF enhanced the mycorrhization and plant growth, and the helper bacteria of AMF could be used as microbial inoculations for plant growth promotion (Xavier and Germida 2003; Bidondo et al. 2016). Further understanding the interactions between the AMF and these helper bacteria would help to reveal the promoting mechanism.

**Table 2:** Effect of spore-associated bacterial isolates on the growth of alfalfa and colonization by *G. margarita*

Bacterial isolates	Fresh weight of stem/leaf (g/pot)	Dry weight of stem/leaf (g/pot)	Fresh weight of roots (g/pot)	Colonization (%)
M060706-5	1.40a	0.32ab	0.68a	36.4a
M060824-7	1.51a	0.34a	0.49ab	29.9ab
M060824-8	1.27ab	0.30ab	0.42b	31.2ab
M061122-2	1.24ab	0.31ab	0.41b	28.0b
M061122-5b	1.15b	0.30ab	0.58ab	31.1ab
M061122-10	1.41a	0.35a	0.53ab	36.7a
M061122-12	1.22ab	0.27ab	0.46b	30.6ab
CK	1.10b	0.25b	0.43b	24.7b

Bacterial isolates M060706-5, M060824-7, M060824-8, M061122-2, M061122-5b, M061122-10 and M061122-12 were mostly related to *Ensifer adhaerens*, *Curtobacterium luteum, Mycobacterium mucogenicum*, *Paenibacillus glycanilyticus*, *Cupriavidus pauculus*, *Bacillus bataviensis* and *Brevibacillus agri*, respectively. Each value is the average of five replications

*CK* control without inoculation

Data with different letters in the same columns are significantly different by DMRT tests at *P* < 0.05

## Electronic supplementary material

Below is the link to the electronic supplementary material.

Supplementary material 1 (DOCX 244 kb)
