# Supplementary material for: Population and function analysis of cultivable bacteria associated with spores of arbuscular mycorrhizal fungus Gigaspora margarita
Source: 3 Biotech. 2017 Apr 8;7(1):8. doi: 10.1007/s13205-017-0612-1 (PMC5385182; doi:10.1007/s13205-017-0612-1)
Supplement: Supplementary file 1 — Supplementary material 1 (DOCX 244 kb) [file 13205_2017_612_MOESM1_ESM.docx]

**Supplementary materials**

**Table S1** Effect of spore-associated bacterial isolates numbered M060706 on the spore germination of *G. margarita* after 14 days culture

| Isolate | Amount of spores | Percent of germination (%) |
| --- | --- | --- |
| M060706-1b | 21 | 28.6c |
| M060706-2 | 21 | 85.7a |
| M060706-3 | 24 | 50.0b |
| M060706-4 | 24 | 50.0b |
| M060706-5 | 15 | 100.0a |
| M060706-6 | 24 | 87.5a |
| M060706-7b | 15 | 20.0c |
| M060706-8 | 15 | 60.0b |
| M060706-9 | 15 | 80.0a |
| M060706-10 | 18 | 33.3c |
| CK | 24 | 50.0b |

Note: Each value is the average of four repeats. Data with different letters in the same column are significantly different by DMRT tests at *P* < 0.05. The same as below.

**Table S2** Effect of spore-associated bacterial isolates numbered M060824 on the spore germination of *G. margarita* after 14 days culture

| Isolate | Amount of spores | Percent of germination (%) |
| --- | --- | --- |
| M060824-1b | 21 | 57.1ab |
| M060824-2 | 21 | 57.1ab |
| M060824-3 | 21 | 28.6c |
| M060824-4 | 27 | 44.4b |
| M060824-5b | 18 | 33.3b |
| M060824-6 | 30 | 30.0c |
| M060824-7 | 27 | 88.9a |
| M060824-8 | 27 | 77.8a |
| M060824-9 | 21 | 71.4a |
| M060824-10 | 21 | 42.9b |
| M060824-11 | 27 | 55.6ab |
| CK | 24 | 50.0b |

**Table S3** Effect of spore-associated bacterial isolates numbered M061017 on the spore germination of *G. margarita* after 14 days culture

| Isolate | Amount of spores | Percent of germination (%) |
| --- | --- | --- |
| M061017-1a | 24 | 62.5b |
| M061017-1b | 21 | 85.7ab |
| M061017-2 | 27 | 55.6b |
| M061017-3 | 27 | 44.4b |
| M061017-4 | 24 | 62.5b |
| M061017-5 | 30 | 80.0ab |
| M061017-6 | 24 | 100.0a |
| M061017-7 | 27 | 77.8ab |
| M061017-8 | 24 | 75.0ab |
| M061017-9 | 21 | 47.6b |
| Control | 27 | 55.6b |

**Table S4** Effect of spore-associated bacterial isolates numbered M061122 on the spore germination of *G. margarita* after 14 days culture

| Isolate | Amount of spores | Percent of germination (%) |
| --- | --- | --- |
| M061122-1 | 21 | 28.6b |
| M061122-2 | 18 | 66.7a |
| M061122-3 | 21 | 42.9ab |
| M061122-4 | 18 | 50.0ab |
| M061122-5b | 18 | 66.7a |
| M061122-6 | 15 | 60.0a |
| M061122-7 | 21 | 42.9ab |
| M061122-8 | 21 | 42.9ab |
| M061122-9 | 18 | 50.0a |
| M061122-10 | 18 | 66.7a |
| M061122-11 | 18 | 33.3b |
| M061122-12 | 18 | 50.0ab |
| Control | 18 | 33.3b |

**Table S5** The most related species of the spore-associated bacteria isolates of *G. margarita*, and their functional evaluation at the aspects of effect on the fungal spore germination, P solution, Chitin decomposing and antagonistic activity**.**

| Isolates | The most related species (%) | Spore  germination | P-  solution | Chitin-  degraded | Antagonistic tests | | |
| --- | --- | --- | --- | --- | --- | --- | --- |
|  |  |  |  |  | *E. coli* | *S. aureus* | *F. oxysporum* |
| M060706-1b | *Mitsuaria chitosanitabida*/98 | 0 | 0 | 4.0 | 0 | 0 | ND |
| M060706-2 | *Leifsonia shinshuensis* /99 | 2 | 6.5 | 0 | 0 | 0 | 0 |
| M060706-3 | *Leifsonia shinshuensis* /98 | 1 | ND | 0 | 0 | 0 | 0 |
| M060706-4 | *Leifsonia shinshuensis*/99 | 1 | 5.3 | 0 | 0 | 0 | 0 |
| M060706-5 | *Ensifer adhaerens*/99 | 2 | ND | ND | 0 | 0 | 0 |
| M060706-6 | *Pseudomonas stutzeri* /99 | 2 | 6.0 | 0 | 0 | 0 | 0 |
| M060706-7b | *Bacillus benzoevorans*/99 | 0 | 4.1 | 0 | 0 | 0 | 0 |
| M060706-8 | *Streptomyces olivochromogenes/*99 | 1 | 4.7 | 0 | 0 | 0 | 0 |
| M060706-9 | *Streptomyces cellulosae* /100 | 2 | 2.0 | 0.9 | 0 | 0 | 0 |
| M060706-10 | *Bacillus arbutinivorans*/99 | 0 | 3.8 | 0 | 0 | 0 | 0 |
| M060824-1b | *Aquitalea magnusonii* /97 | 1 | 3.0 | 0 | 0 | 0 | 0 |
| M060824-2 | *Aquitalea magnusonii* /98 | 1 | 1.8 | 0 | 0 | 0 | 0 |
| M060824-3 | *Arthrobacter ureafaciens*/99 | 0 | 0 | 0 | 0 | 0 | 0 |
| M060824-4 | *Aquitalea magnusonii* /98 | 1 | 1.0 | 0 | 0 | 0 | 0 |
| M060824-5b | *Aquitalea magnusonii* /99 | 1 | 4.0 | 0 | 0 | 0 | 0 |
| M060824-6 | *Aquitalea* *magnusonii* /99 | 0 | 2.2 | 0 | 0 | 0 | 0 |
| M060824-7 | *Curtobacterium luteum*/99 | 2 | 2.7 | 2.1 | 0 | 0 | 0 |
| M060824-8 | *Mycobacterium mucogenicum*/99 | 2 | 0 | 0 | 0 | 0 | 0 |
| M060824-9 | *Ralstonia pickettii* /99 | 2 | 11.3 | 0 | 0 | 0 | 0 |
| M060824-10 | *Arthrobacter ureafaciens* /99 | 1 | 0 | 0 | 0 | 0 | 0 |
| M060824-11 | *Gordonia polyisoprenivoran*/99 | 1 | 0 | 0 | 0 | 0 | 0 |
| M061017-1a | *Lysobacter gummosus*/98 | 1 | 0 | 0 | 5.0 | 4.8 | 0 |
| M061017-1b | *Burkholderia* *mallei* /96 | 1 | 0.8 | 0 | 0 | 0 | 0 |
| M061017-2 | *Achromobacter xylosoxidans*/99 | 1 | 0 | 0 | 0 | 0 | 0 |
| M061017-3 | *Proteus mirabilis*/99 | 1 | 5.7 | 0 | 0 | 0 | 0 |
| M061017-4 | *Bosea thiooxidans* /99 | 1 | 0 | 0 | 0 | 0 | 0 |
| M061017-5 | *Paenibacillus telluris* /94 | 1 | 2.8 | 0 | 0 | 0 | 0 |
| M061017-6 | *Paenibacillus* glycanilyticus/98 | 2 | 3.3 | 5.7 | 0 | 0 | 0 |
| M061017-7 | *Bacillus megaterium* /99 | 1 | 2.3 | 0 | 0 | 0 | 0 |
| M061017-8 | *Nocardia nova*/97 | 1 | 0 | 0 | 0 | 0 | 0 |
| M061017-9 | *Streptomyces roseogriseus*/99 | 1 | 0 | 0 | 0 | 0 | 0 |
| M061122-1 | *Cupriavidus pauculus*/99 | 1 | 0 | 0 | 0 | 0 | ND |
| M061122-2 | *Paenibacillus* glycanilyticus/99 | 2 | 2.3 | 0 | 0 | 0 | 0 |
| M061122-3 | *Amycolatopsis rifamycinica*/98 | 1 | ND | ND | 0 | 0 | ND |
| M061122-4 | *Bacillus muralis*/98 | 1 | 1.7 | 0 | 1.2 | 0 | 0 |
| M061122-5b | *Cupriavidus pauculus*/99 | 2 | 0 | ND | 0 | 0 | ND |
| M061122-6 | *Paenibacillus* glycanilyticus/99 | 2 | 2 | 1.5 | 0 | 0 | 0 |
| M061122-7 | *Streptomyces haeopurpureus*/99 | 1 | 0 | 2.2 | 0 | 0 | 0 |
| M061122-8 | *Rhizobium tropici*/98 | 1 | 0 | 0 | 0 | 0 | 0 |
| M061122-9 | *Nocardia asteroids*/99 | 2 | 0 | 0 | 0 | 0 | 0 |
| M061122-10 | *Bacillus bataviensis* /99 | 2 | 7.7 | 0 | 0 | 0 | 0 |
| M061122-11 | *Cupriavidus pauculus*/99 | 1 | 0 | 0 | 0 | 0 | ND |
| M061122-12 | *Brevibacillus agri* /96 | 1 | 0 | 0 | 0 | 0 | 0 |

Note: Bacteria isolated from the groups of AS (alfalfa in sand/soil pot), AV (alfalfa in vermiculite pot), GS (grain sorghum in sand/soil pot) and MS (maize in sand/soil pot) were numbered as M060706, M060824, M061017 and M061122, respectively. Scores in the third column were assigned as follows: inhibited germination, 0; no effect, 1; promoted germination, 2. ND, no determined, for reason of the isolate could not grow on the test plates. Numbers in the fourth to eighth columns indicated the width (in millimeter) of hydrolysis zone or inhibition zone produced by the tested strains.

**Figure legend**

**Figure S1** The quantities of bacteria isolated from the newly-formed *G. margarita* spores under different subculturing conditions. For each “plant-substrate” system, fitly mature and healthy *G. margarita* spores were used to isolate bacteria by spread TSA plate method. The bacterial CFU (colony formed unit) per plate was enumerated after incubated at 26 °C for 4 days. AS, alfalfa in sand/soil pot; AV, alfalfa in vermiculite pot. GS, grain sorghum in sand/soil pot; MS, maize in sand/soil pot. Each data is the average of three replications. Different letters above the error bars indicate significant differences by DMRT test at *P* < 0.05.

**Figure S2** Representative 16S rRNA gene based tree (Kimura’s correction model; neighbor-joining method, Mega 4.0 software) illustrating the position of the spore-associated bacterial isolates belonged to phyla Proteobacteria (a), Firmicutes (b) and Actinobacteria (c), respectively. Bootstrap values of below 50 are not shown.


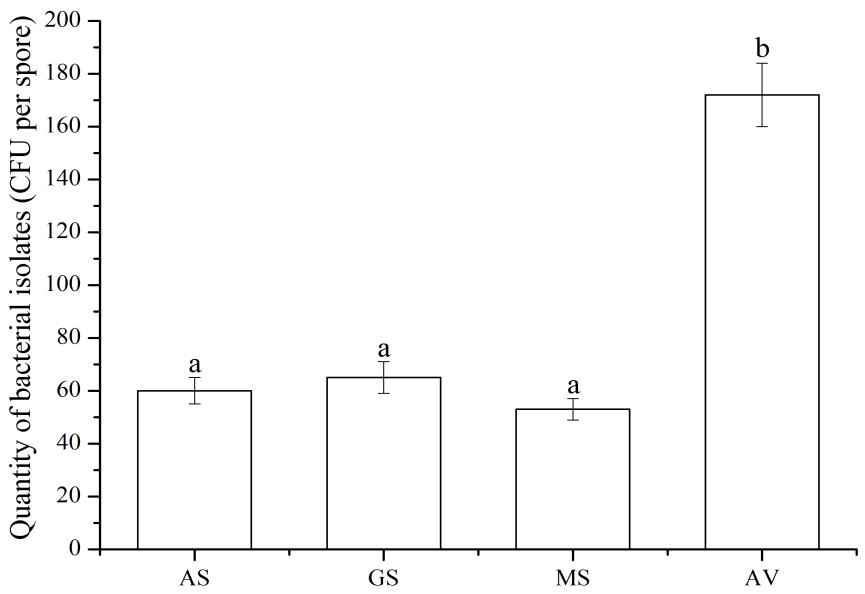


**Figure S1**

M060824-9 (EU589416)

*Ralstonia pickettii* (AY268180.1)

M061017-1b (EU589420)

*Ralstonia eutropha* (AF027407.1)

M061122-11 (EU072715)

M061122-5b (EU072710)

M061017-1a (EU589419)

*Lysobacter gummosus* (DQ065753.1)

M061017-2 (EU589421)

*Achromobacter xylosoxidans* (AF531768.1)

M060706-1b (EU589400)

*Mitsuaria chitosanitabida* (AM501442.1)

M060824-2 (EU589411)

M060824-6 (EU589415)

M060824-1b (EU589410)

*Aquitalea magnusonii* (EU661705.1)

M060824-5b (EU589414)

M060824-4 (EU589413)

M061017-3 (EU589422)

*Proteus mirabilis* (EF091150.1)

M061017-4 (EU589423)

*Bosea thiooxidans*

(AF508803.1)

M061122-8 (EU072713)

*Rhizobium tropici* (EU488756.1)

*Ensifer adhaerens* (EF198418.1)

M060706-5 (EU589404)

89

86

100

100

100

99

99

99

85

55

82

63

63

57

89

0.01

*Burkholderia thailandensis* (EF535235.1)

M061122-1 (EU072706)

M060706-6 (EU589405)

*Pseudomonas balearica* (AF054936.1)

**Figure S2a**

*Curtobacterium luteum* (NR 026157.1)

*Leifsonia shinshuensis* (AB244485.1)

*Streptomyces olivochromogenes* (EF486452.1)

100

99

99

99

89

99

71

98

93

93

88

67

97

74

53

99

0.05

*Arthrobacter ureafaciens* (FN433020.1)

M060824-3 (EU589412)

M060824-10 (EU589417)

M060824-7 (EU072704)

M060706-4 (EU589403)

M060706-2 (EU589401)

M060706-3 (EU589402)

M060706-9 (EU589408)

*Streptomyces cellulosae* (DQ442495.1)

M061017-9 (EU589408)

M061122-7 (EU072712)

*Streptomyces phaeopurpureus* (EU593562.1)

*Streptomyces roseogriseus* (DQ026651.1)

M060706-8 (EU589407)

M061122-3 (EU072708)

*Amycolatopsis rifamycinica* (AY083603.1)

M060824-8 (EU072705)

M060824-11 (EU589418)

*Gordonia polyisoprenivorans* (DQ154925.1)

M061017-8 (EU589427)

M061122-9 (EU072717)

*Nocardia asteroids* (GQ376161.1)

*Mycobacterium mucogenicum* (AY457073.1)

**Figure S2b**

M060706-10 (EU589409)

*Bacillus arbutinivorans* (AF519469.1)

M061122-10 (EU072714)

*Bacillus drentensis* (DQ275176.1)

M060706-7b (EU589406)

M061017-7 (EU589426)

*Bacillus megaterium* (FJ174652.1)

*Bacillus muralis* (AJ628748.1)

M061122-4 (EU072709)

M061122-12 (EU072716)

*Brevibacillus agri* (AB112716.1)

M061017-5 (EU589424)

M061122-2 (EU072707)

*Paenibacillus glycanilyticus* (NR 024759.1)

M061017-6 (EU589425)

M061122-6 (EU072711)

100

100

88

100

99

62

54

100

100

85

100

79

91

0.01

**Figure S2c**
